# Co-infection of SARS-CoV-2 with *Chlamydia* or *Mycoplasma pneumoniae*: a case series and review of the literature

**DOI:** 10.1007/s15010-020-01483-8

**Published:** 2020-07-28

**Authors:** Alessandra Oliva, G. Siccardi, A. Migliarini, F. Cancelli, M. Carnevalini, M. D’Andria, I. Attilia, V. C. Danese, V. Cecchetti, R. Romiti, G. Ceccarelli, C. M. Mastroianni, P. Palange, M. Venditti

**Affiliations:** 1 Department of Public Health and Infectious Diseases, Sapienza University of Rome, Piazzale Aldo Moro 5, 00185 Rome, Italy; 2 Department of Translational and Precision Medicine, Sapienza University of Rome, Rome, Italy; 3 Department of Clinical, Internal, Anestesiology and Cardiovascular Sciences, Sapienza University of Rome, Rome, Italy

**Keywords:** SARS-CoV-2 infection, COVID-19, *Mycoplasma pneumoniae*, *Chlamydia pneumoniae*, Co-infection

## Abstract

**Introduction:**

The novel coronavirus SARS-CoV-2 has spread all over the world causing a global pandemic and representing a great medical challenge. Nowadays, there is limited knowledge on the rate of co-infections with other respiratory pathogens, with viral co-infection being the most representative agents. Co-infection with *Mycoplasma pneumoniae* has been described both in adults and pediatrics whereas only two cases of *Chlamydia pneumoniae* have been reported in a large US study so far.

**Methods:**

In the present report, we describe a series of seven patients where co-infection with *C. pneumoniae* (*n* = 5) or *M. pneumoniae* (*n* = 2) and SARS-CoV-2 was detected in a large teaching hospital in Rome.

**Results and conclusion:**

An extensive review of the updated literature regarding the co-infection between SARS-CoV-2 and these atypical pathogens is also performed.

**Electronic supplementary material:**

The online version of this article (10.1007/s15010-020-01483-8) contains supplementary material, which is available to authorized users.

## Introduction

The novel coronavirus (now called SARS-CoV-2) initially discovered in Wuhan, China, has spread all over the world causing a global pandemic and representing a great medical challenge in terms of treatment, prevention and, not less important, diagnosis [1].

So far, there is limited knowledge on the rate of co-infections with other respiratory pathogens [2]. Likewise, data regarding bloodstream and respiratory bacterial and fungal infections among patients with COVID-19 are very scarce and generally overlooked [3].

While early reports from China and Spain described co-infection as a rare event (6/104, 5.7% and 3/103, 2.9%, respectively) [4, 5], Kim et al. reported the presence of one or more additional pathogen in 24 of 116 patients (20.6%) diagnosed with SARS-CoV-2 infection [6] whereas another study from China showed that up to 80% of SARS-CoV-2 infected subjects had IgM positivity against at least one respiratory agent, therefore highlighting how the detection of other respiratory pathogens cannot be used to rule out COVID-19 diagnosis [7]. Furthermore, it is still unknown whether co-infection with other pathogens, and in particular with intracellular atypical microorganisms, might play a role in determining the prognosis of SARS-CoV-2 infection.

In all the cases, viruses were the most representative agents [2–9]; on the other hand, scarce was the co-infection rate with *Mycoplasma pneumoniae* [10–16] and, interestingly, only two cases out of 1996 with *Chlamydia pneumoniae* has been described so far [17].

Herein, we describe patients where co-infection with *Chlamydia pneumoniae* or *Mycoplasma pneumoniae* and SARS-CoV-2 was detected in our teaching hospital in Rome, Italy. Furthermore, the updated literature regarding the co-infection between SARS-CoV-2 and these atypical pathogens is reviewed.

## Cases description

We retrospectively analyzed data from clinical reports of all the patients admitted to Azienda Ospedaliero-Universitaria Policlinico Umberto I (Sapienza University) of Rome between 1 March and 30 April 2020 with documented SARS-CoV-2 infection. The study was approved by the local Ethics Committee (ID Prot. 109/2020). A total of 182 subjects were tested also for *C. pneumoniae* and *M. pneumoniae*. We found that seven patients (3.8%) were co-infected with SARS-CoV-2 and atypical microorganisms (five *C. pneumoniae*, 2.7%*,* two *M. pneumoniae*, 1.1%). Diagnosis of *C. pneumoniae* and *M. pneumoniae* infection was made based on the serologies (DIESSE Diagnostica Senese S.p.A., sensitivity 97.4% and 94.7%, specificity 94.1% and 92.6% for *C. pneumoniae* and *M. pneumoniae*, respectively) [18] whereas SARS-CoV-2 diagnosis was based on nasopharyngeal swab positivity by using polymerase chain reaction (PCR) [19]. Definition of pneumonia or severe pneumonia was based on the WHO interim guidance and included clinical signs of pneumonia (fever, cough, dyspnoea, fast breathing) with or without signs of severe pneumonia such as respiratory rate > 30 breaths/min, severe respiratory distress, or SpO2 < 90% on room air [19, 20].

Clinical and laboratory characteristics of patients are listed in Table 1, radiological findings are shown in Supplementary Figure 1. Among the patients, four were male and three female, the median age was 73 years (IQR 45–79). All but one patient underwent CT-scan of the lungs, one patient underwent only chest X-ray, which showed bilateral interstitial involvement. Lung CT-scan showed multifocal, bilateral and prevalent peripheral infiltrates in six patients (85.7%), ground glass in five patients (71.4%), subpleural consolidation in four patients (57.1%). No patient had pleural effusion. According to guidelines [19], severe pneumonia was observed in 2/7 (28.5%) cases. All patients underwent therapy with hydroxychloroquine and azithromycin, 5/7 with heparin (57.1%), 3/7 with corticosteroids (42.8%), 2/7 with lopinavir/ritonavir (28.5%), 2/7 with tocilizumab (28.5%). One patient received also teicoplanin that has been described as potentially active against coronaviruses [21, 22]. As for oxygen delivery, two patients (28.5%) received high-oxygen non-invasive support (one high-flow nasal cannula, one C-PAP), three (43.0%) were on Venturi masks and the remaining two (28.5%) were on room air. All patients were discharged after a median length of hospitalization of 28 days (IQR 13–34).

**Table 1:** Characteristics of patients with SARS-CoV-2 and *Chlamydia pneumoniae* (*n* = 5) or *Mycoplasma pneumoniae* (*n* = 2) co-infection

Pt	Age/sex	Comorbidities	Clinical presentation	Type of co-infection	Laboratory findings on admission	Oxygen delivery	Therapy	ICU/death	Lenght of hospitalization, days
Pt#1	86/F	Hypertension, diabetes	Fever, altered mental status	*M. pneumoniae*	WBC 4850 N/L 2820/1330 PLT 198,000 CRP 0.25 LDH 207 D-dimer 4473	Room air	Hydroxychloroquine, azithromycin, heparin	No/No	32
Pt#2	19/M	None	Fever, cough	*M. pneumoniae*	WBC 5250 N/L 4470/520 PLT 127,000 CRP 11.01 LDH 556 D-dimer 383	C-PAP and high-flow nasal cannula	Hydroxychloroquine, azithromycin, teicoplanin, tocilizumab, corticosteroid, heparin, piperacillin/tazobactam	Yes/No	41
Pt#3	73/F	Congestive heart failure, bronchial asthma, chronic renal failure	Fever, cough, shortness of breath, fatigue	*C. pneumoniae*	WBC 4850 N/L 46,560/1740 PLT 223,000 CRP 10.05 LDH 308 D-dimer 4473	Venturi mask	Lopinavir/ritonavir, hydroxychloroquine, azithromycin, heparin, piperacillin/tazobactam	No/No	21
Pt#4	45/F	None	Fever, shortness of breath, chest pain	*C. pneumoniae*	WBC 7590 N/L 4240/2470 PLT 208,000 CRP 0.16 LDH 158 D-dimer 234	Room air	Hydroxychloroquine, azithromycin, corticosteroid, heparin	No/No	13
Pt#5	77/M	Hypertension, diabetes	Fever, myalgia	*C. pneumoniae*	WBC 7390 N/L 6240/700 PLT 206,000 CRP 9.4 LDH 416 D-dimer 3170	C-PAP	Hydroxychloroquine, azithromycin, tocilizumab, corticosteroid, ceftriaxone	No/No	28
Pt#6	79/M	Congestive heart failure, bronchial asthma	Shortness of breath	*C. pneumoniae*	WBC 16,170 N/L 14,310/1130 PLT 76,000 CRP 0.51 LDH 371 D-dimer 4382	Venturi mask	Hydroxychloroquine, azithromycin	No/No	34
Pt#7	60/M	None	Fever, cough	*C. pneumoniae*	WBC 8440 N/L 7260/840 PLT 216,000 CRP 3.27 LDH 239 D-dimer 581	Venturi mask	Lopinavir/ritonavir, hydroxychloroquine, azithromycin	No/No	7

*ICU* intensive care unit, *WBC* white blood cell, *N* neutrophils, *L* lymphocytes, *PLT* platelets, *CRP* C-reactive protein

Finally, when clinical outcomes (ICU admission and intra-hospital mortality) of 175 patients without *M. pneumoniae* or *C. pneumoniae* co-infection [median age 63 years (IQR 52–76), 71 (40.5%) females] were compared to those with co-infection, no differences were observed [1/7 (14.2%) vs. 24/175 (13.7%) and 0/7 (0%) vs. 25/175 (14.2%), respectively].

## Discussion and review of the literature

In the present report we described for the first time in Europe [2, 17] that patients with SARS-CoV-2 infection might be co-infected, among agents of atypical pneumonia, not only with *M. pneumoniae* but also with *C. pneumoniae.* These microorganisms can affect adults and children, are usually mild and only occasionally could represent life-threatening conditions. In particular, *M. pneumoniae* may cause epidemics and spread in close clusters. As the majority of symptomatic patients with SARS-CoV-2 infection develop an atypical pneumonia syndrome with fever, cough, and shortness of breath, co-infections with *C. pneumoniae* or *M. pneumoniae* are likely obscured, making therefore difficult the differential diagnosis only based on clinical presentation [19, 20]. The rate of co-infection with *M*. *pneumoniae* in SARS-CoV-2 pneumonia patients has been reported in the literature [10–16] whereas co-infection with *C. pneumoniae* has been reported only in two cases in a large US study involving 5700 patients with COVID-19 [17] (Table 2). In detail, Fan et al. described a case of a 36-year old male requiring Intensive Care Unit (ICU) admission and presenting with severe lymphopenia, low platelet count and cold agglutinin titer of 1:8 with *M. pneumoniae* antibody titer of 1:160 [14] whereas Ziang Gao et al. described a case of 49-year old female presenting with cough, expectoration and lung CT scan showing multiple ground-glass opacities in bilateral lower lobes [16]. Gayam et al. reported that six out of 350 patients (1.71%) with SARS-CoV-2 infection were also diagnosed with *M. pneumoniae* detected by serology [12] and, in a recent double-center Chinese study conducted at Qingdao and Wuhan regions and involving 68 patients with SARS-CoV-2 infection, the authors found a not-negligible rate of co-infection with common respiratory pathogens, with 8/68 (11.7%) of subjects showing also *M*. *pneumoniae* positive serology [7]. In the same study, a highly different distribution between the two regions (7/30, 23.3%, in Qingdao and 1/38, 2.63%, in Wuhan) was observed [7]. Although the whole rate of co-infection was far different, retrospective studies conducted in Spain and in the UK showed a similar number of SARS-CoV-2-*M. pneumoniae* co-infection (0.97% and 1.49%, respectively), the latter detected with multiplex PCR assays [5, 11]. In pediatric patients, co-infection with *M. pneumoniae* was surprisingly high, accounting for 16/34 (47.0%) of the total and a case report described the presence of COVID-19 infection with pleural effusion complicated by secondary *M. pneumoniae* infection in a 12-year old boy [13, 15]. As for *C. pneumoniae*, only one large US study which had the aim to describe the clinical characteristics and outcomes of 5700 hospitalized patients with COVID-19 found two *C. pneumoniae* cases out of 42/1996 positive samples tested also for respiratory pathogens panel [17]. Of note, and unlike our report, no clinical information of these two cases of *C. pneumoniae* and SARS-CoV-2 co-infection were available [17].

**Table 2:** Literature data on SARS-CoV-2 and *Mycoplasma pneumoniae/Chlamydia pneumoniae* co-infection

Author	Type of study	Type of patients	Overall rate of co-infection	Type of *M. pneumoniae* or *C. pneumoniae* co-infection	Diagnostic method of co-infection	Number of patients with *M. pneumoniae* or *C. pneumoniae* co-infection	Outcome
Blasco et al. [4]	Retrospective study in patients with SARS-CoV-2 infection at Clinic University Hospital of Valencia	Adults	3/103 (2.9%)	*M. pneumoniae*	Multiplex PCR assay	1/103 (0.97%) *M. pneumoniae*	NA
Xing et al. [6]	Double-centre study in China (Qingdao and Wuhan regions) in patients with SARS-CoV-2 infection	Adults	25/68 (36.7%) 24/30 (80%) Qingdao 1/38 (2.63%) Wuhan	*M. pneumoniae*	Serology	8/68 (11.7%) *M. pneumoniae* 7/30 (23.3%) Qingdao 1/38 (2.63%) Wuhan	NA
Easom et al. [10]	First 68 patients with SARS-CoV-2 infection at a Regional Infectious Diseases Unit (RIDU) in the UK	Adults	29/67 (43.2%)	*M. pneumoniae*	Multiplex PCR assay	1/67 (1.49%) *M. pneumoniae*	NA
Zhang et al. [9]	Hospitalized patients with SARS-CoV-2 infection in No. 7 Hospital of Wuhan	Adults	7/58 (12.0%)	*M. pneumoniae*	Serology	5/58 (8.6%) *M. pneumoniae*	NA
Wu et al. [12]	Pediatric patients with laboratory-confirmed COVID-19 at Qingdao Women’s and Children’s Hospital and Wuhan Children’s Hospital	Pediatrics	19/34 (55.88%)	*M. pneumoniae*	Multiplex PCR assay	16/34 (47.0%) *M. pneumoniae* *M. pneumoniae* alone = 11; *M. pneumoniae* + RSV = 2 *M. pneumoniae* + EBV = 2 *M. pneumoniae* + RSV + InfluenzaA/B = 1	Survived
Gayam et al. [11]	Out of 350 patients hospitalized with SARS-CoV-2 infection at Interfaith Medical Center, Brooklyn, New York, a series of six patients with co-infection from SARS-CoV-2 and *M. pneumoniae*	Adults	6/350 (1.71%)	*M. pneumoniae*	Serology	6/350 (1.71%) *M. pneumoniae*	1/6 (16.6%) ICU admission and death
Fan et al. [13]	Case report	Adult (36-year old male)	NA	*M. pneumoniae*	Cold agglutinin titer of 1:8 with a *M. pneumoniae *antibody titer of 1:160	NA	ICU admission
Gao et al. [15]	Case report	Adult (49-year old female)	NA	*M. pneumoniae*	Serology	NA	Recovery
Chen et al. [14]	Case report	Pediatric (12-year-old boy)	NA	*M. pneumoniae*	Serology	NA	Recovery
Richardson et al. [16]	All consecutive hospitalized patients with confirmed severe acute respiratory syndrome coronavirus 2 (SARS-CoV-2) at any of 12 Northwell Health acute care hospitals between March 1, 2020 and April 4, 2020	Adults	42/1996 (2.1%)	*C. pneumoniae*	Respiratory pathogens panel	2/42 (4.76%) *C. pneumoniae*	NA

*ICU* intensive care unit, *RSV* respiratory syncytial virus, *EBV* Ebstein–Barr virus

Similarly to what has been reported in the literature, the majority of our patients presented with fever, cough and/or shortness of breath, showed bilateral infiltrates at the lung CT, received oxygen support and were treated with hydroxychloroquine and azithromycin.

The possible co-existence of pathogens other than SARS-CoV-2 in patients with COVID-19 infection focuses the attention on the real incidence of SARS-CoV-2 and other bacterial/viral or even fungal co-infections, which should be investigated to find whether co-infections might play a role in disease severity and/or mortality [2]. In our case series, only one patient needed ICU admission, no patients died and the median duration of hospitalization was 28 days.

The present report has several limitations. First, not all the hospitalized patients with SARS-CoV-2 infection were tested also for *C*. *pneumoniae* and *M*. *pneumoniae*; therefore, we could present only a part of patients with serological detection of atypical pathogens and infection with SARS-CoV-2 and the real incidence of co-infection cannot be truly established, requiring the need of testing always for pathogens other than SARS-CoV-2. Then, for the diagnosis of co-infections we could rely only on serology, since molecular analyses of respiratory samples specifically detecting *M*. *pneumoniae* or *C. pneumoniae* were lacking. In fact, although rarely, serology might be limited by possible false positive results, which should always be taken into account when deciding to exclude SARS-CoV-2 infection. One additional limitation is represented by the lack of paired samples to confirm prior serological results for the diagnosis of atypical pathogens.

However, with these limitations in mind, we reported for the first time the clinical characteristics of patients with *C. pneumoniae,* and not only *M. pneumoniae,* as a co-existing pathogen during SARS-CoV-2 infection. Therefore, the present report opens the path to additional studies investigating the real incidence of co-infections during SARS-CoV-2 epidemic and their possible impact on infection severity and mortality. Not less important, keeping in mind that in the future SARS-CoV-2 might be sporadic and not the cause of a pandemic infection anymore, we could infer that the serological detection of these atypical pulmonary pathogens in subjects presenting with respiratory symptoms cannot be used to rule out a diagnosis of COVID-19 [2, 4, 7, 23]. On the other hand, the reliability of serology for atypical bacteria should be considered when excluding the diagnosis of COVID-19 in patients with nasopharyngeal negative swabs (which has been demonstrated to occur in a not-negligible percentage of cases) [24], symptoms highly suggestive of SARS-CoV-2 infection and positive serology for other pathogens. Based on these considerations, physicians should assume that the presence of a pathogen other than SARS-CoV-2 does not ensure that a subject does not have also COVID-19.

In conclusion, SARS-CoV-2 infection might be associated with other common respiratory pathogens, including those causing atypical pneumonia. This finding should be considered in the near future, especially when ruling out the diagnosis of COVID-19. Therefore, the search for SARS-CoV-2 infection should be added to routine diagnostic testing even though other common respiratory pathogens are detected. Further studies are needed to evaluate the possible influence of co-infections on the severity of SARS-CoV-2 infection.

## Data availability statement

The data used to support the findings of this study are available from the corresponding author upon request.

## Electronic supplementary material

Below is the link to the electronic supplementary material.

Supplementary file1 (DOCX 941 kb)
