# Supplementary material for: Co-infection of SARS-CoV-2 with Chlamydia or Mycoplasma pneumoniae: a case series and review of the literature
Source: Infection. 2020 Jul 28;48(6):871–7. doi: 10.1007/s15010-020-01483-8 (PMC7386385; doi:10.1007/s15010-020-01483-8)
Supplement: Supplementary file 1 — Supplementary file1 (DOCX 941 kb) [file 15010_2020_1483_MOESM1_ESM.docx]

Patient 1 Patient 2


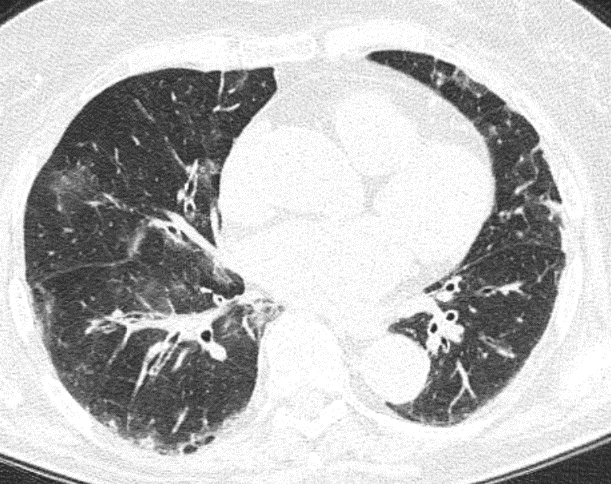

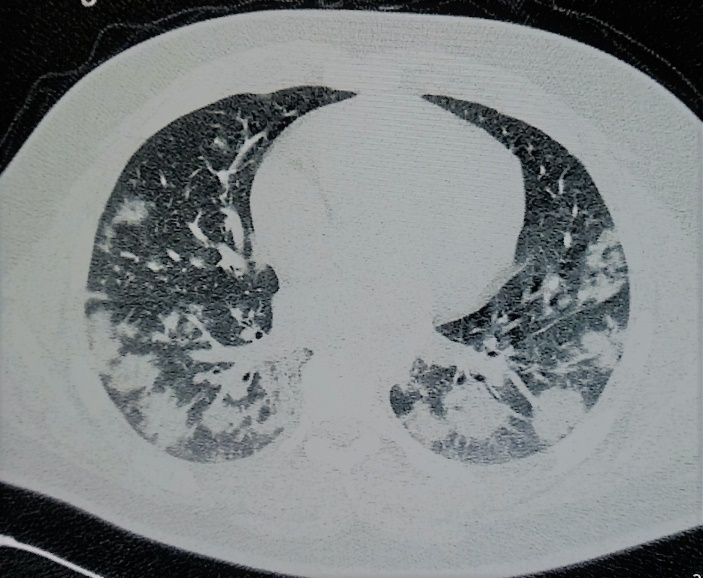


Patient 3 Patient 4


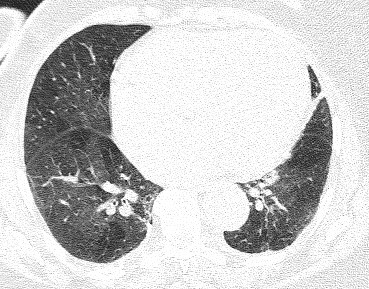

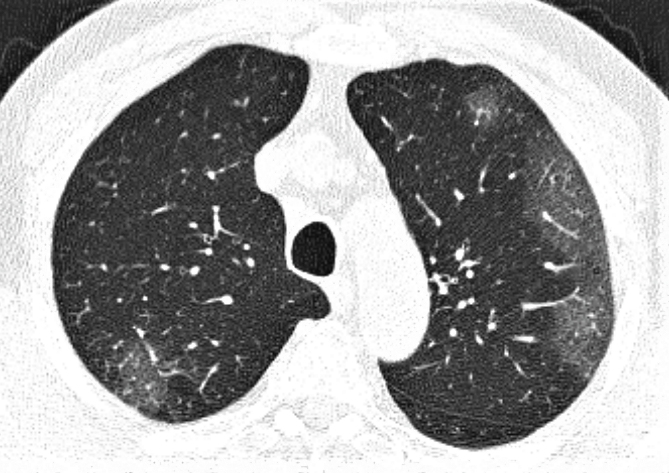


Patient 5 Patient 6


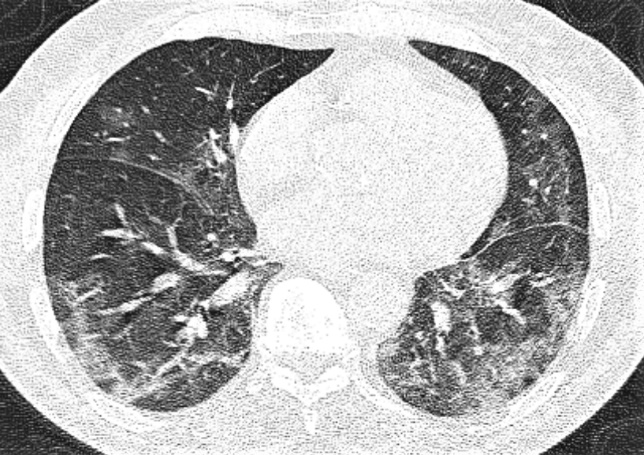

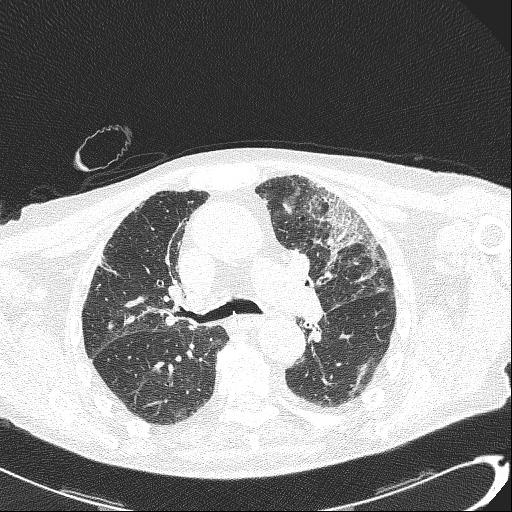


Patient 7


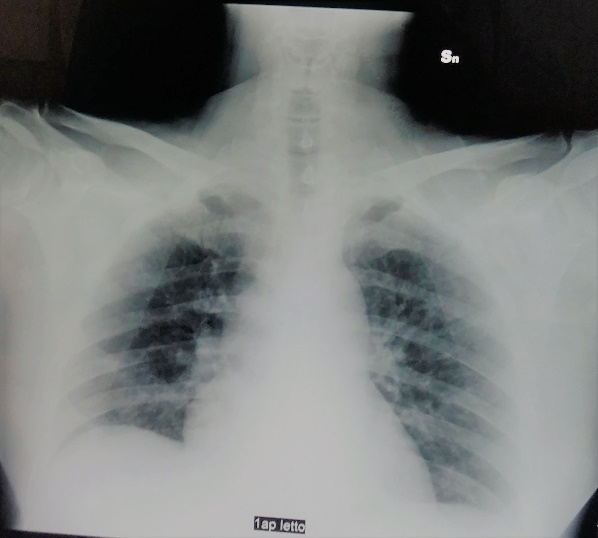


**Supplementary Figure1.** Radiological findings of patients with SARS-CoV-2 and *Chlamydia pneumoniae* (n=5) or *Mycoplasma pneumoniae* (n=2) co-infection.
